# Brain plasticity associated with prolonged shooting training: a multimodal neuroimaging investigation from a cross-sectional study

**DOI:** 10.3389/fnhum.2025.1530642

**Published:** 2025-02-12

**Authors:** Keying Zhang, Tao Zhao, Yu Ding, Jia Cheng, Chunmei Cao

**Affiliations:** ^1^Department of Physical Education, Southeast University, Nanjing, China; ^2^Division of Sports Science and Physical Education, Tsinghua University, Beijing, China; ^3^School of Psychology, Beijing Language and Culture University, Beijing, China; ^4^Department of Mechanical Engineering, Tsinghua University, Beijing, China

**Keywords:** shooting training, MRI, functional connectivity, cortical thickness, structural connectivity

## Abstract

**Background:**

Although training has been recognized as a potential contributor to neuroplasticity in athletes, the impact of prolonged shooting training on human brain plasticity remains unclear in the existing literature.

**Methods:**

In this cross-sectional study, we used a multimodal neuroimaging analysis, including the analysis of functional blood oxygenation level-dependent (BOLD) magnetic resonance imaging (MRI) images, structural T1-weighted MRI images, and diffusion MRI images, to systematically identify differences between elite shooters and normal controls.

**Results:**

The results showed that compared to male normal controls, male elite shooters had higher regional homogeneity (ReHo) in the frontal lobe, parietal lobe, precuneus, thalamus, and cingulate gyrus, as well as higher functional connectivity between the medial frontal cortex (MedFC) and temporooccipital middle temporal gyrus (toMTG). Male elite shooters also showed higher cortical thickness in the right inferior temporal lobe; lower fractional anisotropy (FA) values in the right superior longitudinal fasciculus (SLF), right inferior fronto-occipital fasciculus (IFF), and right anterior thalamic radiation (ATR); lower axial diffusivity (AD) value in forceps minor and left ATR; and lower structural connectivity between right putamen and right inferior parietal cortex (IPC), right IPC and right paracentral cortex, and right paracentral cortex and right superior parietal cortex (SPC).

**Conclusion:**

Elite male shooters exhibited optimized resting-state functional activity, functional connectivity, and morphological features compared to normal controls. Prolonged shooting training may contribute to enhancing the brain’s functional and structural plasticity related to motor control, attentional focus, and emotion regulation in male shooters; however, similar changes have not been observed in female shooters.

## 1 Introduction

Brain plasticity is the brain’s ability to adapt and reorganize its function and structure in response to environmental changes (Bonfanti et al., 2021; Li et al., 2024; Kolb et al., 2003). Enhancing brain plasticity to mitigate cognitive decline and improve brain function is a major research focus. Evidence shows that exercise can enhance both functional and structural brain plasticity (Cui et al., 2019). For example, physical training can lead to detectable changes in the gray matter within weeks (Taubert et al., 2012). Twelve weeks of balance training induces structural plasticity in areas such as the superior temporal cortex, visual association cortex, posterior cingulate cortex, and superior frontal sulcus (Taubert et al., 2012). Additionally, regular aerobic exercise over 6 weeks has been shown to increase anterior hippocampal volume (Thomas et al., 2016). Active exercise may act centrally in the brain, promoting the release of brain-derived neurotrophic factor (BDNF), which in turn enhances cognitive performance and neuroplasticity (El-Sayes et al., 2019). In particular, a study by Perini et al. (2016) found that a single bout of aerobic exercise significantly facilitated learning mechanisms within visual and motor domains, suggesting that physical activity may promote brain plasticity in a short period of time. Furthermore, a randomized controlled trial by Deodato et al. (2024) showed that a dual-task protocol had a more evident effect on neurophysiological outcomes in people with migraine, indicating that complex tasks involving both physical and cognitive challenges can also impact brain plasticity. These discoveries emphasize the significant role of physical activity in promoting brain plasticity, offering insights into the mechanisms through which exercise can improve central nervous system function and cognitive resilience. While these studies have explored the effects of physical training on brain plasticity, there is a lack of research systematically comparing high-level shooters with control groups to investigate the neural characteristics associated with long-term shooting training.

Shooting requires balance and stability, and performance is influenced by postural balance, muscle tremor, and gun stability (Ihalainen et al., 2016; Park et al., 2019). Advances in techniques such as EEG and MRI allow non-invasive observation of brain reorganization. EEG studies reveal neurophysiological differences between elite and novice shooters, both during aiming and at rest. Elite shooters show increased alpha and beta power in the occipital and parietal lobes and heightened theta power in the frontal lobe compared to novices (Duffy, 2017). They also exhibit lower event-related desynchronization (ERD) in the alpha band and less cortical activation in the left central–temporal–parietal lobe, indicating greater neural efficiency (Del Percio et al., 2009). Additionally, elite shooters demonstrate stronger theta power in the anterior cingulate and medial frontal cortex, areas associated with focused attention (Doppelmayr et al., 2008). Resting-state studies show higher functional connectivity in the left temporal, left-posterior temporal, left frontal, left-central, and right-parietal lobes among elite shooters, as well as greater network-clustering coefficients and small-world characteristics in theta and low-alpha bands (Gong et al., 2019). However, research using MRI to investigate brain plasticity induced by shooting training is limited. For example, 4 weeks of intensive first-person shooting (FPS) training increased cortical thickness in the bilateral parahippocampal cortex, somatosensory cortex, superior parietal lobule, and right insula (Momi et al., 2018). This study also reported improved cognitive function and increased resting-state functional connectivity between the left thalamus and left parahippocampal gyrus (Momi et al., 2021). Unlike FPS training, real-world shooting involves long-term training, making it challenging to track neural changes over time. Therefore, comparing neural differences between shooting athletes and non-trained controls using MRI is crucial for understanding brain plasticity associated with long-term shooting training.

Studies investigating brain plasticity induced by shooting training using MRI techniques are limited. A study by Momi et al. (2018) found that 4 weeks of intensive first-person shooting (FPS) training led to increased cortical thickness in the bilateral parahippocampal cortex (PHC), somatosensory cortex (S1), superior parietal lobule (SPL), and right insula. In a follow-up study, Momi et al. (2021) observed that 4 weeks of FPS training not only improved gamers’ cognitive function but also increased their resting-state functional connectivity between the left thalamus and left parahippocampal gyrus. However, unlike the relatively short duration of FPS game training, real-world shooting training lasts for years or even decades, making it challenging to track neural changes over time as has been performed in FPS studies. To address this gap, the present study aimed to compare the neural differences between long-term shooting athletes and non-athlete controls, a comparison that has not been previously explored. Therefore, we compare elite shooting athletes with non-athlete controls who have no prior experience with firearms or regular sports training. This comparison aims to reveal the neural differences associated with long-term shooting training. To achieve this, we used multimodal MRI images including functional blood oxygenation level-dependent (BOLD) MRI images, structural T1-weighted MRI images, and diffusion MRI images, to investigate the brain differences between elite shooters and normal controls.

Therefore, this cross-sectional study aims to investigate the functional and structural brain plasticity induced by long-term shooting training by analyzing functional, structural, and diffusion MRI images of elite shooting athletes and normal controls. The hypotheses for this study were as follows: (1) there are functional and structural brain differences between elite shooters and controls; (2) these differences are associated with shooting-related functions, including motor control, attentional focus, and emotion regulation; and (3) in addition, the manifestation of neuroplasticity may differ between male and female athletes.

## 2 Materials and methods

### 2.1 Participants

A total of 35 participants were recruited for this cross-sectional study, comprising 11 Chinese national shooting team athletes (age: 22.1 ± 3.0; 5 males and 6 females, see Table 1 for details) with extensive shooting training experience of 10.3 ± 3.0 years, and 24 college students (age: 19.8 ± 2.0; 16 males and 8 females) who had no prior exposure to firearms in real-world scenarios and lacked regular sports training experience. All participants were right-handed, reported normal or corrected-to-normal vision, and met the requirements for magnetic resonance imaging scans. Participants were tested at separate time intervals and were unaware of group assignments, thereby reducing observer and performance bias. To account for potential sex-based differences in brain plasticity, as observed in previous studies (Luders et al., 2006; Lv et al., 2010; Kawachi et al., 2002), we chose to analyze male and female athletes separately. This approach allowed for a more precise examination of the sex-specific neural adaptations to shooting training. For male participants, the elite group consisted of 5 male participants, while the control group comprised 16 male participants. The independent samples *t*-test revealed no significant age difference between the two groups (EG: 21.2 ± 1.8 years vs. CG: 20.8 ± 1.8 years, *t* = 0.428, *p* = 0.673). For female participants, the elite group consisted of six male participants, while the control group comprised eight male participants. There is a significant age difference between the two groups (EG: 22.8 ± 3.8 years vs. CG: 17.9 ± 0.6 years, *t* = 3.704, *p* = 0.003). Therefore, to mitigate the impact of age differences on experimental outcomes, age was considered a covariate in the statistical analysis (Sowell et al., 2003). Subjects were recruited in October 2021, with experimental data collected between November and December 2021. This study was approved by the ethics committee. The written informed consent forms were signed by all participants before MRI scanning.

**Table 1:** Demographic data and athletic performance of shooters.

Subject	Sex	Age	Training years	Specialties	Personal best records
S1	Female	21	13	10-m air rifle	National University Shooting Championship Champion
S2	Female	20	9	10-m air pistol	World University Shooting Championship Team Silver Medal
S3	Female	27	15	50-m rifle three positions	National Shooting Championship Runner-up
S4	Female	18	5	10-m air pistol	National University Shooting Championship Champion
S5	Female	24	11	10-m air rifle	National Games Team Champion
S6	Female	27	15	50-m rifle three positions	World University Games Team Champion
S7	Male	23	9	10-m air rifle	World University Games Team Champion
S8	Male	21	8	10-m air rifle	National Games Mixed Team Silver Medal, Asian Air Rifle Championship Junior Group Champion
S9	Male	20	9	10-m air pistol	World Shooting Championship Junior Team Silver Medal
S10	Male	23	10	10-m air pistol	World University Shooting Championship Runner-up
S11	Male	19	9	10-m air rifle	National University Shooting Championship Champion

### 2.2 MRI data acquisition

Functional, structural, and diffusion MRI images were acquired using a 3 Tesla Philips Achieva scanner using a 32-channel head coil. To minimize information bias, standardized MRI protocols for data collection and analysis were performed. The functional images were acquired as 240 contiguous echo planar imaging (EPI) functional volumes with a voxel resolution of $2.875\times 2.875\times 3.5\;mm^{3}$ (TR = 2 s, TE = 30 ms, flip angle = 90°, FOV = $230\times 230\;mm^{2}$ , number of slices = 37). The structural images were obtained using a T1-weighted, 3D, magnetization-prepared, rapid-acquisition, gradient echo (MPRAGE) volume acquisition whose voxel resolution was $1\times 1\times 1\;mm^{3}$ (TR = 7.5 ms, TE = 3.7 ms, flip angle = 8°, FOV = $230\times 230\;mm^{2}$ ). The diffusion tensor imaging (DTI) images were obtained with a gradient-weighted spin-echo sequence with a voxel resolution of $1.75\times 1.75\times 2.8\;mm^{3}$ (TR = 5,534 ms, TE = 85 ms, flip angle = 90°, FOV = $224\times 224\;mm^{2}$ , number of slices = 50). During MRI scanning, all participants were asked to lie still on the scanner and close their eyes trying to think nothing but avoid falling asleep. A foam head holder and padding were placed around participants’ heads to decrease their head motion and earplugs were offered to participants to shield them from noise.

### 2.3 Resting-state functional metrics analysis

Resting-state functional metrics analysis was carried out using the Data Processing Assistant for Resting-State fMRI (DPARSF 5.1) toolbox^1^ (Yan et al., 2017), which is based on Statistical Parametric Mapping 12 (SPM 12) software.^2^ The first 10 time points of fMRI images were removed to reduce the influence of magnetization instability at the beginning, and slice timing correction was performed for temporal inconsistency. Then, images of each subject were realigned using rigid body transformation to do motion correction. Functional and T1-weighted images were all reoriented manually and scalp stripped using the BET module of FMRIB Software Library (FSL 6.0) software^3^ (Jenkinson et al., 2012). Then, T1-weighted images were co-registered to the functional space. After coregistration, segmentation was performed into three parts: white matter, gray matter, and cerebrospinal fluid (CSF). Nuisance covariates regression was conducted with white matter, CSF, and Friston 24 parameters (Satterthwaite et al., 2013). Considering global signal regression (GSR) is very controversial, this study analyzed results with and without GSR. Finally, images were normalized into MNI space using DARTEL and resliced to a $3\times 3\times 3\;mm^{3}$ voxel size (Ashburner, 2007).

After preprocessing, two males in the control group were excluded because of excessive head motion. The exclusion criteria were a maximum head motion of 2.5 mm and 2.5 degrees for all groups. For males, the elite group remained the same, while the control group included 14 males (age: 21.00±1.80 ) after head motion correction. No significant difference in age was found between the two groups (*t* = 0.214, *p* = 0.833). For females, both groups remained the same. Then, regional homogeneity (ReHo) values were calculated for all groups. ReHo was used to assess brain regional synchronization. Kendall’s coefficient of concordance (KCC) was used to measure the ReHo of the time series of a voxel with those of its nearest neighbors in a voxel-wise way (Zang et al., 2004).

The results were smoothed with a Gaussian kernel of 10 mm full width at half maximum (FWHM). A two-sample *t*-test was performed to compare the differences in ReHo between elite shooters and normal controls, including mean FD_Jenkinson value (head motion metric) and age as covariates across all groups (Jenkinson et al., 2002). Multiple comparison correction was conducted using a permutation test (5,000 permutations in the present study) with threshold-free cluster enhancement (TFCE), which was proved to reach a high balance between family-wise error rate and test–retest reliability/replicability (Chen et al., 2018; Winkler et al., 2016).

Volume-based temporal dynamic analysis was performed with the ReHo metric to evaluate intra-individual temporal variation using DPABI (Yan et al., 2017). First, sliding hamming windows (length: 30 TR, stride: 1 TR) were applied to functional images. Then mean and standard deviation (SD) maps across time windows were calculated to characterize temporal dynamic resting-state metrics. SD results were regarded as the metric of temporal variation and were smoothed with a Gaussian kernel of 4 mm FWHM. A two-sample *t*-test was performed to compare dynamic temporal variation between elite shooters and normal controls, including mean FD_Jenkinson value (head motion metric) and age as covariates across all groups (Jenkinson et al., 2002). The multiple comparison correction method was the same as above using a permutation test (5,000 permutations) with TFCE.

### 2.4 Resting-state functional connectivity analysis

The resting-state functional MRI images were preprocessed and calculated using the CONN functional connectivity toolbox 20.b^4^ (Whitfield-Gabrieli and Nieto-Castanon, 2012). For preprocessing, the default preprocessing pipeline for volume-based MNI space analyses was chosen. Motion estimation and correction were first performed on functional images. Then functional and structural images were all centered to (0,0,0) coordinates. Slice timing correction was performed on functional images to correct for inter-slice differences in acquisition time. Next, functional outlier detection was performed using ART-based identification of outlier scans for scrubbing. Intermediate scrubbing settings (97th percentiles in normative sample) with a global signal *Z*-value difference threshold of 5 and subject-motion threshold of 0.9 mm were used. Simultaneous gray/white/CSF segmentation and MNI normalization were executed for functional images and structural images with default tissue probability maps used. Functional images were then smoothed using a Gaussian kernel of 8 mm FWHM. Two males in the control group were excluded because of excessive head motion, following the same DPARSF preprocessing procedure as described above.

After preprocessing was finished, the denoising procedure was followed, which was one of the most important steps for cleaning up the resting-state data. The CompCor method was chosen, including five components for white matter, five for CSF, twelve for realignment parameters, forty-five for scrubbing, and two for the effect of rest (2 components) was chosen. Functional images were then band-pass filtered (0.008–0.09 Hz) to further increase sensitivity.

A region of interest (ROI) to ROI analysis was conducted to compare the resting-state functional connectivity (rs-FC) differences between elite shooters and normal controls. The ROIs included atlas and network level regions, in which the atlas level consists of the FSL Harvard-Oxford atlas (cortical and subcortical areas) and automated anatomical labeling (AAL) atlas (cerebellar), while network-level consists of an atlas of 32 commonly used networks and areas such as default mode network (DMN), MPFC/PCC areas and so on (Destrieux et al., 2010). Pearson’s correlation between the averaged time series of each ROI was computed. The results between the ROIs were corrected for multiple comparisons using a two-sided contrast with a *p*-value <0.05, cluster-level *p*-FDR corrected (MVPA omnibus test) (Chumbley et al., 2010).

A seed-to-voxel analysis was performed after the ROI-to-ROI analysis, in which significant brain regions found in the ReHo analysis were chosen as the seeds. Pearson’s correlation coefficient between the averaged time series of chosen seeds and other voxels in the brain was computed. Cluster-based inferences using the random field theory parametric statistics were performed with cluster threshold *p* < 0.05 and cluster size *p*-FDR correction.

### 2.5 Cortical thickness analysis

Cortical thickness analysis was performed using FreeSurfer 7.1.1 on CentOS 8, which is a robust open-source ecosystem around a Linux platform^5^ (Fischl, 2012). Brain cortical surface reconstruction from the T1-weighted images was conducted with *recon-all* command implemented in FreeSurfer. The *Recon-all* command parcellated participants’ brains according to two atlases: the Desikan–Killiany atlas and the Destrieux atlas (Destrieux et al., 2010). Cortical thickness was measured using the average value of two distances: the distance from a vertex on the white matter surface to the pial surface, and the distance from a point on the pial surface to the white matter surface (Fischl and Dale, 2000). The results showed that there are 148,555 vertices in the left hemisphere and 148,857 vertices in the right hemisphere. The individual images were normalized to a template image called *fsaverage,* which was an average of 40 subjects and re-shaped into a pial surface. A Gaussian kernel of 20 mm FWHM was used to smooth the individual images to improve the inter-individual correspondence in the anatomical surface. In such surface-based analysis, larger smoothing kernels can be used than in volumetric-based analysis, as there is less risk of smoothing across gyri.

A vertex-wise group analysis was then performed to compare the difference in cortical thickness between elites and controls with *mri_glmfit* command. Multiple comparison correction was performed using cluster correction with the *mri_glmfit-sim* command. The vertex-wise cluster threshold was set as a *p*-value of <0.001, and the cluster-wise *p*-threshold was set as a *p*-value of <0.05.

### 2.6 DTI metrics analysis

Preprocessing and calculation of DTI metrics were performed using FSL 6.0 software (see text footnote 3) (Jenkinson et al., 2012) and MRtrix 3.02 software^6^ (Tournier et al., 2019) on CentOS 8 platform. For preprocessing, DTI images were first denoised using the *dwidenoise* command and corrected for motion and eddy currents using the *dwifslpreproc* command. A brain mask was then created by running the *bet2* command on one of the *B* = 0 (no diffusion weighting) images. Finally, the diffusion tensor model was fitted using the *dtifit* command to calculate maps for fractional anisotropy (FA) and axial diffusivity (AD, first eigenvalue L1). FA measures the directional dependence of diffusion, reflecting fiber density and coherence within a voxel, whereas AD measures water diffusion parallel to the axon fibers (Guerrero et al., 2023).

Tract-based spatial statistics (TBSS 1.2) were performed to conduct a voxel-wise analysis of the diffusion maps. A standardized pipeline for TBSS analysis recommended in the FSL user guide website^7^ was used. First, all FA images were non-linearly registered to a standard FA template. The mean FA image was then created and skeletonized. Subsequently, all FA images of the subjects were projected onto the mean FA skeleton with a threshold of 0.2. These projection steps onto the FA skeleton were also applied to the AD metric. Finally, differences in FA and AD between elite shooters and normal controls were compared. Statistical inference was performed using the TFCE option (5,000 permutations) in the *randomize* command, with a threshold of *p* < 0.05 to correct for multiple comparisons (Nichols and Holmes, 2002).

### 2.7 Structural connectome analysis

A structural connectivity matrix was estimated for each participant using DTI images and T1-weighted images through MRtrix 3.02 software (See text footnote 6) (Tournier et al., 2019), FreeSurfer 7.1.1 software (See text footnote 5) (Fischl, 2012), FSL 6.0 software (See text footnote 3) (Jenkinson et al., 2012), and Advanced Normalization Tools (ANTs 2.3.5).^8^ For preprocessing, DTI images were denoised using a data-driven principal component analysis. Eddy currents and head motion were then corrected using the *dwifslpreproc* command. Bias field correction was performed using the N4 algorithm in ANTs. A mask was also created for following processing steps. Thereafter, a basic function from participants’ DTI data was created using the Dhollander function, which estimated different basis functions for each tissue type. Next, multishell–multitissue constrained spherical deconvolution was performed, and images of the fiber orientation densities (FODs) were overlaid onto the estimated tissues. Finally, the FOD images were normalized to enable comparison between participants. T1-weighted images were then extracted into five tissue categories (gray matter, subcortical gray matter, white matter, cerebrospinal fluid, and pathological tissue). B0 images and tissue maps were acquired and averaged for coregistration. The *fslroi* and *flirt* commands were then used to create a transformation matrix for registration between the tissue map and the B0 images. Following this, a seed region along the gray/white matter boundary was created, and a target streamline count of 10 million was set across the whole brain. Tractography results were filtered with a streamlined count of 1 million using spherical-deconvolution-informed filtering of tractograms via the *tcksift2* command. Finally, quality checks were performed for all steps mentioned above to ensure proper structural connectome construction. T1-weighted images were cortical parcellated and implemented by FreeSurfer 7.1.1 software based on Desikan–Killiany atlas (Fischl, 2012). Finally, a 84×84 symmetrical matrix of streamline counts was extracted for each participant using the FreeSurfer parcellations noted above with *tck2connectome* command.

Whole-brain structural connectome group-wise statistics at the edge level were performed using the network-based statistics (NBS 1.2) toolbox^9^ (Zalesky et al., 2010), which was accompanied by MATLAB 2019b. A permutation test (5,000 permutations) with FDR correction (*p* < 0.05) was used to control for multiple comparisons. BrainNet Viewer 1.7 toolbox was used for the visualization of significant networks^10^ (Xia et al., 2013).

In summary, outcome events and summary measures for ReHo, functional connectivity, cortical thickness, FA intensity, and structural connectivity, will be reported in the following Results section.

## 3 Results

### 3.1 Resting-state functional metrics

**Figure 1:**
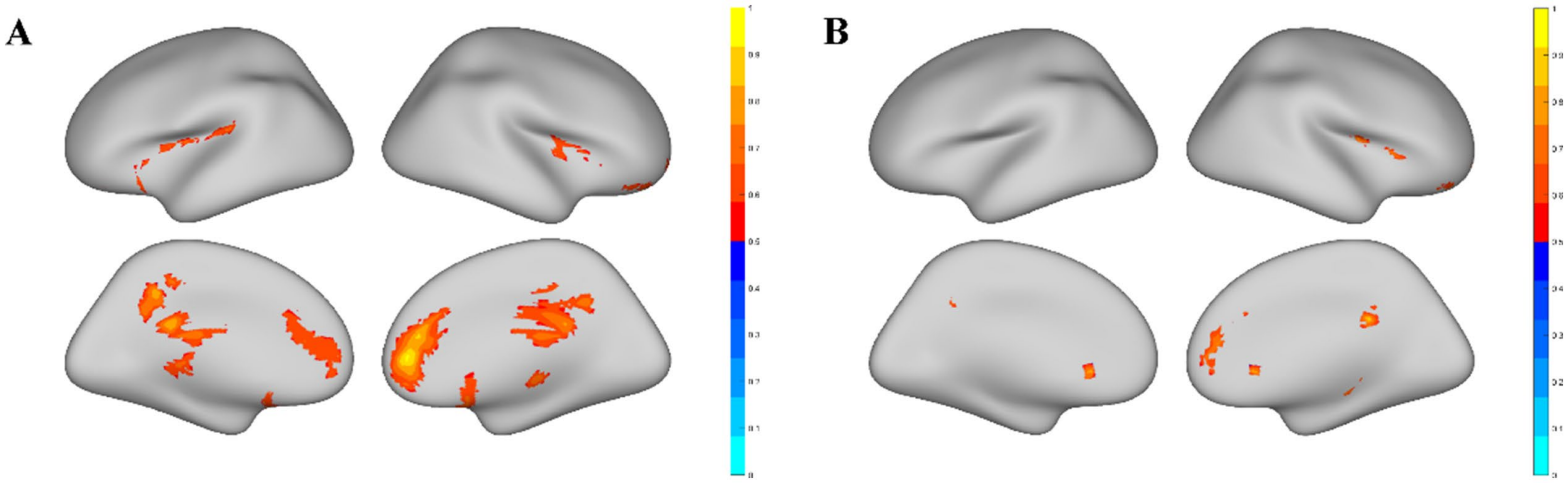
Significant brain areas related to the ReHo metric. **(A)** Brain areas with significant ReHo value differences between male elite shooters and male controls. Compared to male controls, male elite shooters had higher ReHo values in the frontal lobe, limbic lobe, parietal lobe, temporal lobe, anterior cingulate, thalamus, precuneus, insula, cingulate gyrus, and medial frontal gyrus. **(B)** Brain areas with significant temporal dynamic variation differences of ReHo between male elite shooters and male controls. Male elite shooters had higher temporal dynamic variation in the frontal lobe, temporal lobe, limbic lobe, parietal lobe, anterior cingulate, and cingulate gyrus than male controls.

Significant discrepancies in ReHo values were observed between elite shooters and normal controls only in males, while no significant differences were observed in females. The results of the ReHo metric without global signal regression are presented in Figure 1A. Two clusters showed significant ReHo differences between male elite shooters and male controls, and their peak MNI coordinates are distributed in (18, 36, 3) and (18, −42, 30), respectively. Male elite shooters exhibited higher ReHo values than controls in the frontal lobe, parietal lobe, thalamus, precuneus, and cingulate gyrus than male controls (Figure 1A). Male elite shooters had higher volume-based temporal dynamic variation in the frontal lobe, parietal lobe, and cingulate gyrus than male controls (Figure 1B).

### 3.2 Resting-state functional connectivity

**Figure 2:**
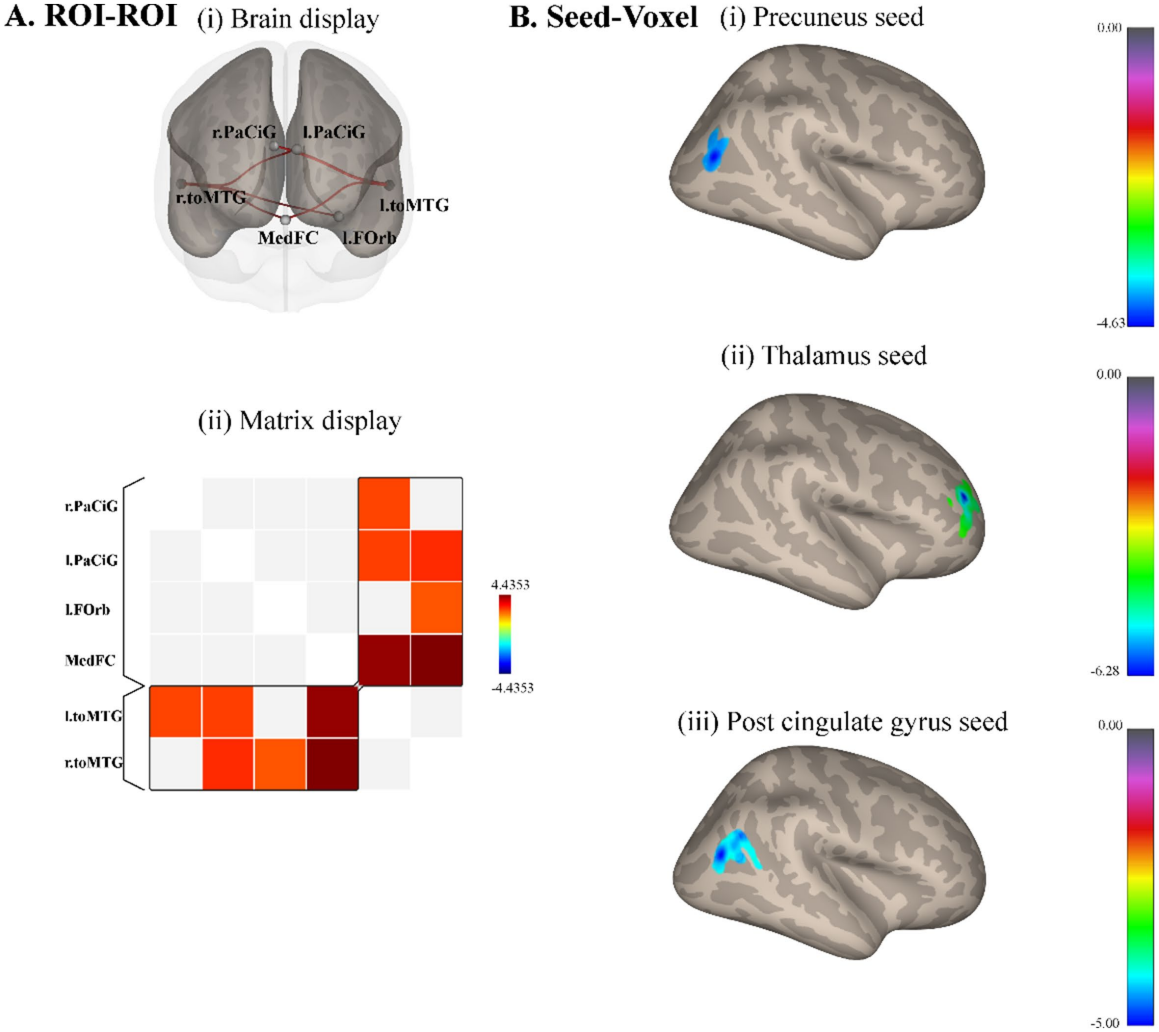
Resting-state functional connectivity differences between male elite shooters and male controls. **(A)** ROI–ROI functional connectivity differences. Compared to male controls, male elites had significantly higher functional connectivity intensity between MedFC and r.toMTG, MedFC, and l.toMTG. (i) Anterior brain display of ROI–ROI analysis. (ii) Brain matrix display of ROI–ROI analysis. **(B)** Seed-voxel functional connectivity differences. (i) Precuneus as the seed, elite shooters had lower functional connectivity between precuneus and r.sLOC. (ii) Thalamus as the seed, elite shooters had lower functional connectivity between thalamus and right frontal pole. (iii) Post-cingulate gyrus as the seed, elite shooters have lower functional connectivity between post-cingulate gyrus and r.sLOC, right angular gyrus. MedFC, Medial frontal cortex. r.toMTG, right temporooccipital middle temporal gyrus. l.toMTG, left temporooccipital middle temporal gyrus. r.sLOC, right superior lateral occipital cortex.

ROI-ROI whole-brain analysis revealed that compared with male controls, male elites showed significantly higher connectivity intensity between medial frontal cortex (MedFC) and right temporooccipital middle temporal gyrus (r.toMTG) (*p* = 0.035, FDR corrected), medial frontal cortex and left temporooccipital middle temporal gyrus (l.toMTG) (*p* = 0.035, FDR corrected), left paracingulate gyrus (l.PaCiG) and r.toMTG (*p* = 0.009, uncorrected), l.PaCiG and l.toMTG (*p* = 0.013, uncorrected), right paracingulate gyrus (r.PaCiG) and l.toMTG (*p* = 0.015, uncorrected), left frontal orbital cortex and r.toMTG (*p* = 0.020, uncorrected) (Figure 2A). The precuneus, thalamus, and cingulate gyrus were analyzed for seed-voxel connectivity based on ReHo results. The precuneus had a lower correlation with the right superior lateral occipital cortex in elite shooters (cluster size = 191, *p* = 0.009, FDR corrected) (Figure 2Bi). The thalamus showed a lower correlation with the right frontal pole (cluster size = 327, *p* = 0.0007, FWE corrected) (Figure 2Bii). While the post-cingulate gyrus had a lower correlation with the right superior lateral occipital cortex (cluster size = 158, *p* = 0.005, FWE corrected).

### 3.3 Cortical thickness

**Figure 3:**
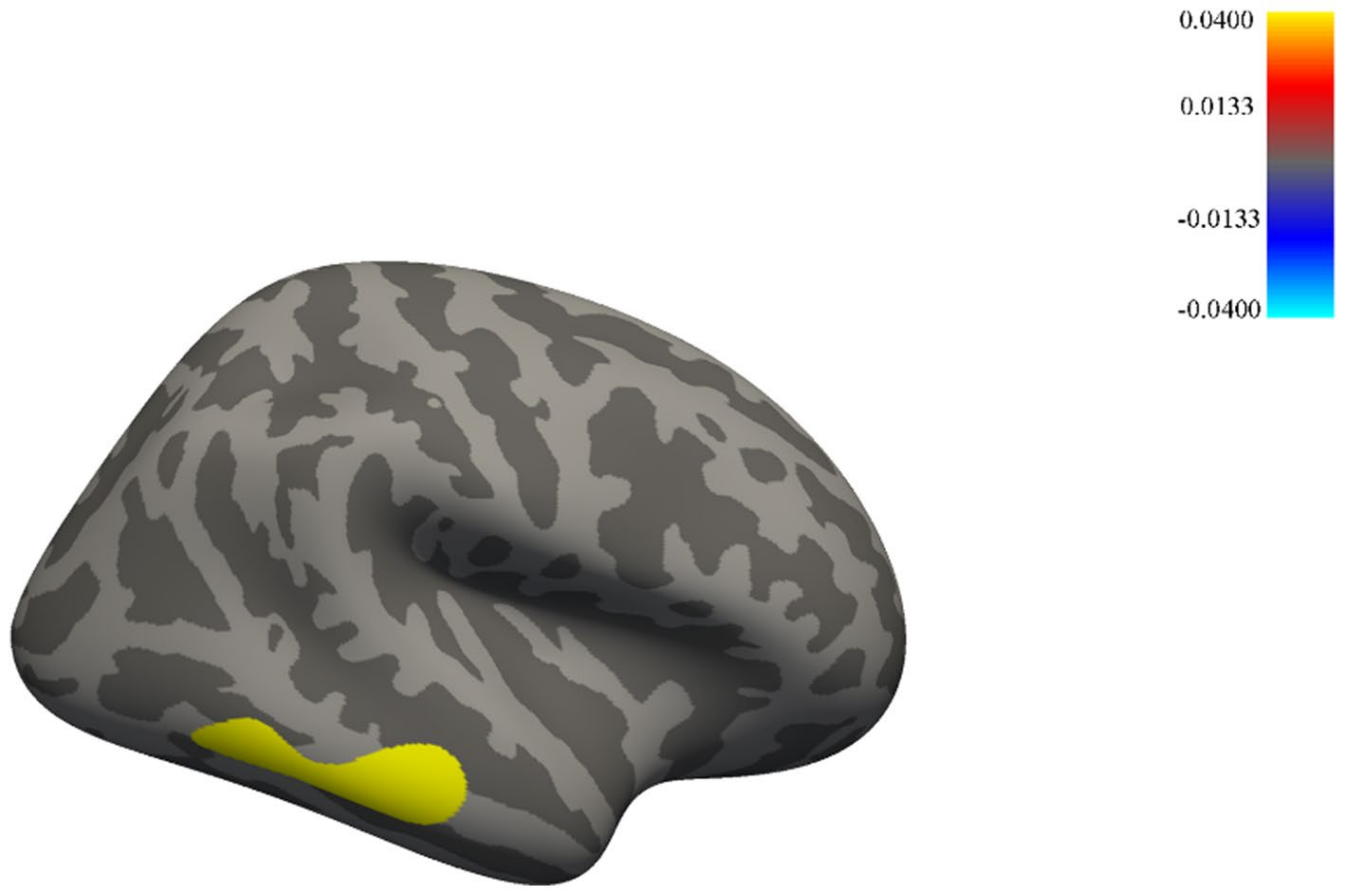
Brain areas with significant cortical thickness differences between male elite shooters and male controls. Male elites had higher cortical thickness in the inferior temporal lobe of the right hemisphere than male controls.

A significant difference in cortical thickness was observed between male elite shooters and male controls. Male elite shooters exhibited higher cortical thickness in the right inferior temporal lobe (*p* = 0.0002) than male normal controls (Figure 3).

### 3.4 DTI metrics

**Figure 4:**
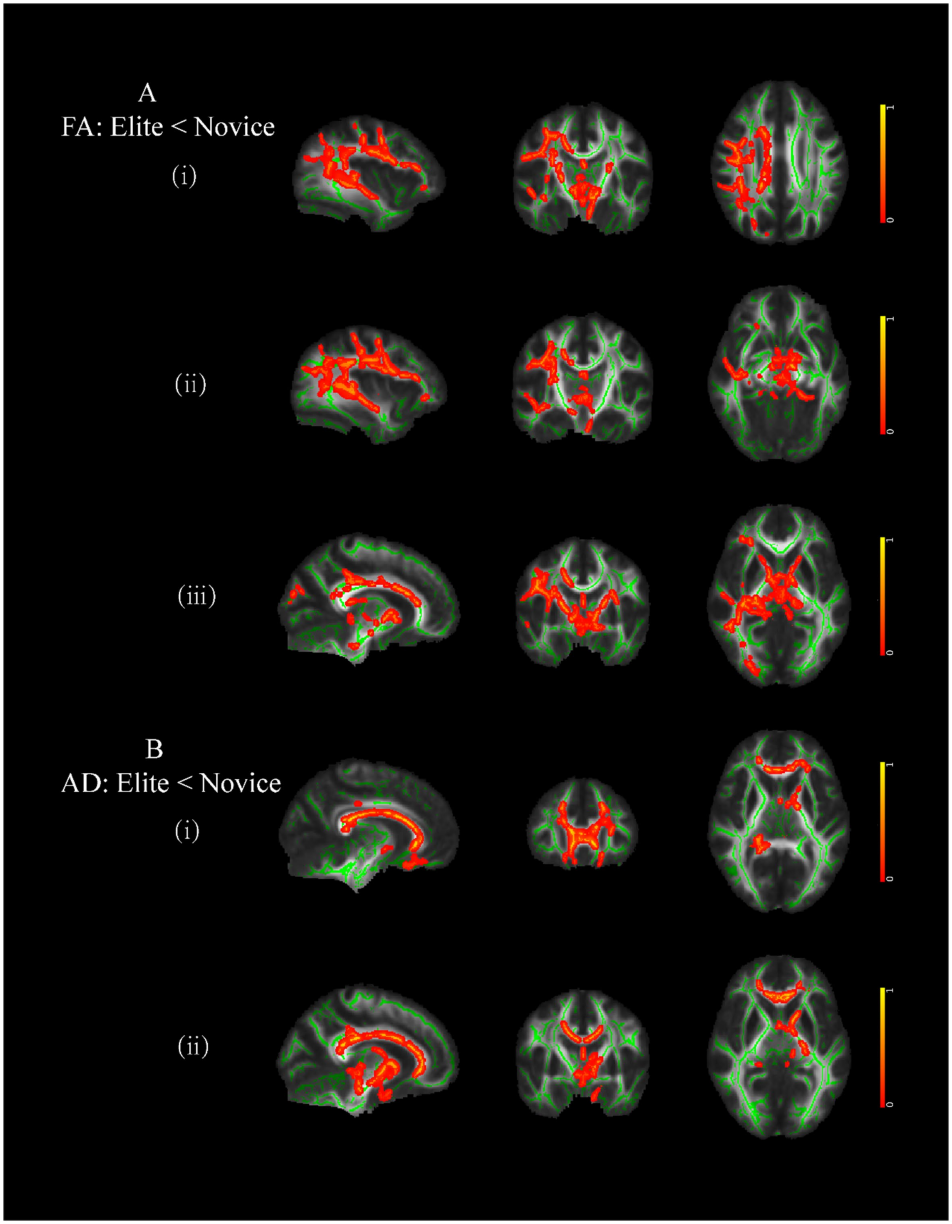
Brain areas of white matter with significant differences between male elite shooters and male controls. **(A)** Compared to male controls, male elites had lower FA intensity in (i) right SLF, (ii) right IFOF, and (iii) right ATR. **(B)** Compared to male controls, male elites had lower AD intensity in (i) forceps minor, and (ii) left ATR. SLF, superior longitudinal fasciculus. IFOF, inferior fronto-occipital fasciculus. ATR, anterior thalamic radiation.

Compared to male controls, male elite shooters had lower FA intensity in the right superior longitudinal fasciculus (SLF), right inferior fronto-occipital fasciculus (IFOF), and right anterior thalamic radiation (ATR) (Figure 4A). In addition, male elite shooters had lower intensity in forceps minor and left anterior thalamic radiation than male controls (Figure 4B).

### 3.5 Structural connectome

**Figure 5:**
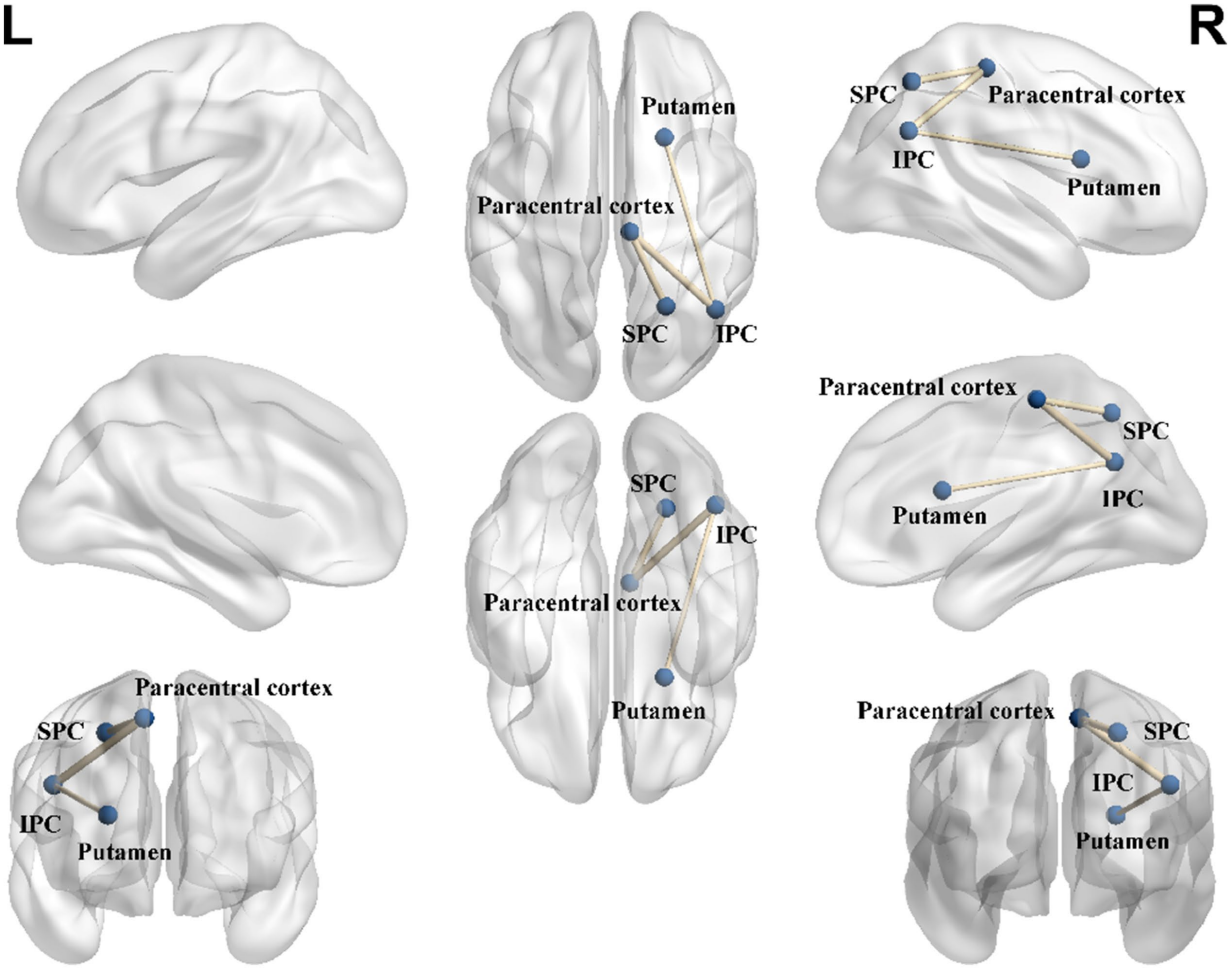
Structural connectome differences between male elite shooters and male controls. Compared to male controls, male elite shooters had lower structural connectivity between right putamen and right IPC, right paracentral cortex and right IPC, and right SPC and right paracentral cortex. IPC, inferior parietal cortex. SPC, superior parietal cortex.

Male elite shooters had lower structural connectivity between right putamen and right inferior parietal cortex (IPC) (*p* < 0.05, FDR corrected), right paracentral cortex and right inferior parietal cortex (*p* < 0.05, FDR corrected), right superior parietal cortex (SPC), and right paracentral cortex (*p* < 0.05, FDR corrected) (Figure 5).

## 4 Discussion

The results of this study indicate that male elite shooters who have been involved in long-term shooting training exhibited functional and structural differences in brain regions associated with shooting-related functions, supporting the experimental hypotheses. Our results were quite consistent with previous studies and extended knowledge of neurophysiological differences between elite shooters and normal controls.

Elite shooters exhibit differences in brain regions including the frontal lobe, parietal lobe, cingulate gyrus, thalamus, and precuneus. The functions associated with these brain areas align with shooting-related abilities. The frontal lobe is involved in a wide range of cognitive functions such as coordinating voluntary movements, emotional expression and regulation, and attentional control (including selective attention). Higher ReHo values in the frontal lobe may be associated with greater motor control and more focused attention in male elite shooters. The parietal lobe is involved in sensory perception and integration (Benoit and Schacter, 2015), coordinating arm and eye movements, and plays a role in attentional coordination (Bisley and Goldberg, 2010), which is crucial for shooting performance. The cingulate gyrus is involved in processing emotions, regulating behaviors, autonomic motor functions, and decision-making, which contribute to high shooting scores (Apps et al., 2016; Bubb et al., 2018; Koob and Volkow, 2010). For example, elite shooters also show stronger theta band power in the anterior cingulate area and medial frontal cortex, which are thought to be involved in focused attention (Doppelmayr et al., 2008). The thalamus performs several functions such as relaying and integrating sensory information, emotional control, hearing and visual pathways, consciousness, and motor control (Zhang et al., 2008). The precuneus is involved in various complex functions, including integrating information related to environmental perception and conscious information processing (Lyu et al., 2021; Dordevic et al., 2022). Therefore, the current results suggest that elite shooters possess greater flexibility in emotional regulation, motor control, and attentional focus, which helps them to quickly adjust their state and maintain optimal performance during shooting competitions.

In addition, the ROI–ROI contrast analysis of male elites minus male controls showed that male elites had higher connectivity intensity between MedFC and toMTG. Higher functional connectivity between MedFC and toMTG may help male elite shooters achieve better control of emotion and regulate their status more effectively, as it allows faster acquisition of sensory information and quicker feedback processing. Seed-voxel contrast analysis of male elite shooters minus male controls showed that male elites had lower connectivity intensity between precuneus and r.sLOC, between thalamus and right frontal pole, between post-cingulate gyrus and r.sLOC, right angular gyrus. The lateral occipital cortex was known to be selective for objects relative to feature-matched controls and played an important role in human object recognition (Appelbaum et al., 2010). The frontal pole was believed to execute the function of monitoring action outcomes (Koechlin, 2011). Lower connectivity intensity between these regions in elite shooters reflected higher neural efficiency in controlling attention and emotions.

Similar results were obtained regarding the brain structure of elite shooters. Elite shooters exhibited differences in cortical thickness in the right inferior temporal gyrus, as well as in the fractional anisotropy (FA) of the right superior longitudinal fasciculus (SLF), right inferior fronto-occipital fasciculus (IFOF), and right arcuate fasciculus (ATR). Additionally, differences were observed in the structural connectivity between the right putamen and right inferior parietal cortex (IPC), right paracentral cortex and right IPC, and right superior parietal cortex (SPC) and right paracentral cortex when compared to the control group. The inferior temporal lobe is involved in the maintenance of short-term emotional, visual, and cognitive memory (Ezzyat et al., 2018; Siegel et al., 2015). This brain area was found to be correlated with shooting-related abilities, suggesting that male elite shooters have enhanced visual recognition ability and better emotional regulation than normal controls. The SLF mainly communicated between the frontal and parietal lobes and interconnected almost all cortical areas of the lateral cerebral hemisphere (Nakajima et al., 2020). The IFOF plays an important role in semantic language processing, goal-oriented behavior, and visual switching tasks (Conner et al., 2018). The ATR is likely to be involved in an emotional/motivational system that helps to mediate separation distress and sadness (Coenen et al., 2012). Less FA in the right SLF, IFOF, and ATR reflected that elite shooters had lower attentional and cognitive-related function levels in the resting state. Less AD in the forceps minor and left ATR implied lower reacting consciousness of elite shooters in the resting state. The putamen was interconnected with many other brain structures to influence motor behaviors, which included motor planning, learning, and execution, specifying amplitudes of movement and movement sequences (Marchand et al., 2008). The IPC integrated various modalities such as somatosensory, visual, and auditory information, and played an important role in various cognitive functions such as selective attention (Bareham et al., 2018). The paracentral cortex was related to visuospatial working memory and attention (Seo et al., 2012). Lower structural connectivity intensity in this network reflected higher neural efficiency in elite shooters, as they are able to achieve better motor control and attentional focus with less effort.

The shooting-related brain plasticity changes observed in this study were only evident in the male population. While previous studies have suggested that brain structure, function, and plasticity exhibit gender-specific characteristics (Luders et al., 2006; Kawachi et al., 2002; Catenaccio et al., 2016), the lack of significant findings in this study among females does not necessarily indicate a limitation. Instead, it highlights a result that contrasts with prior research, which may point to gender differences in brain plasticity or reflect the need for more stringent experimental controls to uncover potential changes in females. Factors such as hormonal influences or menstrual cycle effects on brain plasticity could also play a role, warranting further investigation.

However, the relatively small sample size in the elite athlete group is a clear limitation of this study. The limited availability of high-level athletes, particularly female athletes, may have contributed to the lack of significant results. This issue is common in research involving elite sports populations, as seen in previous studies on brain plasticity in elite athletes. To address this limitation, future research should aim to refine experimental protocols, increase sample sizes, and include more diverse participants to confirm whether gender-specific differences in brain plasticity exist. Expanding the sample size in elite athlete populations would enhance the robustness of the findings and provide more reliable insights into brain plasticity. Furthermore, incorporating both genders in future research, with a larger sample size, could offer a more comprehensive perspective on the effects of shooting training.

In addition, the sources of neural differences between elite shooters and controls remain unclear, as these differences could either preexist or develop over time due to long-term training. Future research could address these issues by including task-based MRI studies, which would provide a more comprehensive understanding of brain plasticity related to long-term shooting training. Additionally, intervention studies utilizing techniques such as transcranial direct current stimulation (tDCS) or transcranial magnetic stimulation (TMS) could be conducted to investigate the underlying neural mechanisms and their potential modulating effects on shooting performance. Furthermore, longitudinal studies could explore the trajectory of neural changes over the course of an athlete’s training career, allowing for a better understanding of the development of shooting-related brain plasticity.

In conclusion, the study indicates that prolonged shooting training may enhance brain functional and structural plasticity related to motor control, attentional focus, and emotion regulation in male shooters, though similar effects have not been observed in female shooters, which may limit the generalizability of the findings. Future research should aim to include diverse gender populations and different training contexts to better understand the broader applicability of these results.

## Data Availability

The raw data supporting the conclusions of this article will be made available by the authors, without undue reservation.

## Footnotes

^1^ http://rfmri.org/DPARSF

^2^ https://www.fil.ion.ucl.ac.uk/spm/

^3^ https://fsl.fmrib.ox.ac.uk/fsl/fslwiki/FSL

^4^ https://www.nitrc.org/projects/conn

^5^ http://surfer.nmr.mgh.harvard.edu/

^6^ https://www.mrtrix.org/download/

^7^ https://fsl.fmrib.ox.ac.uk/fsl/fslwiki/TBSS/UserGuide

^8^ https://github.com/ANTsX/ANTs

^9^ https://www.nitrc.org/projects/nbs

^10^ https://www.nitrc.org/projects/bnv/
